# Intergroup bias in punishing behaviors of adults with autism spectrum disorder

**DOI:** 10.3389/fpsyt.2022.884529

**Published:** 2022-08-19

**Authors:** Chenyu Qian, Shisei Tei, Takashi Itahashi, Yuta Y. Aoki, Haruhisa Ohta, Ryu-ichiro Hashimoto, Motoaki Nakamura, Hidehiko Takahashi, Nobumasa Kato, Junya Fujino

**Affiliations:** ^1^Department of Psychiatry and Behavioral Sciences, Graduate School of Medical and Dental Sciences, Tokyo Medical and Dental University, Tokyo, Japan; ^2^Medical Institute of Developmental Disabilities Research, Showa University, Tokyo, Japan; ^3^Department of Psychiatry, Graduate School of Medicine, Kyoto University, Kyoto, Japan; ^4^Institute of Applied Brain Sciences, Waseda University, Saitama, Japan; ^5^School of Human and Social Sciences, Tokyo International University, Saitama, Japan; ^6^Department of Psychiatry, School of Medicine, Showa University, Tokyo, Japan; ^7^Department of Language Sciences, Graduate School of Humanities, Tokyo Metropolitan University, Tokyo, Japan; ^8^Kanagawa Psychiatric Center, Kanagawa, Japan

**Keywords:** autism spectrum disorder, behavioral economics, decision-making, intergroup bias, third-party punishment

## Abstract

Groups are essential elements of society, and humans, by nature, commonly manifest intergroup bias (i.e., behave more positively toward an ingroup member than toward an outgroup member). Despite the growing evidence of various types of altered decision-making in individuals with autism spectrum disorder (ASD), their behavior under the situation involving group membership remains largely unexplored. By modifying a third-party punishment paradigm, we investigated intergroup bias in individuals with ASD and typical development (TD). In our experiment, participants who were considered as the third party observed a dictator game wherein proposers could decide how to distribute a provided amount of money while receivers could only accept unconditionally. Participants were confronted with two different group situations: the proposer was an ingroup member and the recipient was an outgroup member (IN/OUT condition) or the proposer was an outgroup member and the recipient was an ingroup member (OUT/IN condition). Participants with TD punished proposers more severely when violating social norms in the OUT/IN condition than in IN/OUT condition, indicating that their decisions were influenced by the intergroup context. This intergroup bias was attenuated in individuals with ASD. Our findings deepen the understanding of altered decision-making and socioeconomic behaviors in individuals with ASD.

## Introduction

The hallmark of human behavior is the tendency to create social groups (1). We often communicate more deeply with people who share similar interests, identities, and beliefs to established nations, religions, and political parties. This tendency cultivates intergroup bias, which can further facilitate people to act more positively toward an ingroup member than toward an outgroup member (1–4). For example, we tend to cooperate more with ingroup and less with outgroup members (1, 3). Moreover, we tend to punish outgroup members more strongly when they committed norm violations than ingroup members (2, 4). Intergroup bias has long been explored in various academic approaches, including psychology, politics, and recently, cognitive neuroscience (1–4).

Intergroup bias might emerge differently among individuals with autism spectrum disorder (ASD), who are characterized by altered social interaction and atypical interests (5–10). Previous studies have suggested that individuals with ASD tend to be less sensitive to context stimuli, and make more rational and/or consistent decisions (11–16). For example, loss/gain framing effects have been reported to be lower when individuals with ASD make choices between gambles (13, 17). Farmer et al. (14) showed that individuals with ASD were less influenced by decoy options in an attraction effect task. Moreover, our recent research has also supported such attenuated cognitive bias in ASD; we showed that the sunk cost effect was lower in individuals with ASD (sunk cost is defined as the tendency to continue an investment, or take an action, although future costs are larger than benefits, if costs of time, money, or effort were previously incurred) (15, 16). Choice consistency is regarded normative in the conventional economic theory (13–16); therefore, these previous findings contribute to a better understanding of ASD by showing a valuable strength and difficulty in decision-making of individuals with ASD (18–20).

Given that groups are central to lives and the people inherently tend to exhibit intergroup bias (21–23), it is productive to study response of individuals with ASD in interpersonal situations involving group membership; this can deepen the understanding of their behavior. However, to the best of our knowledge, only one recent study has examined intergroup bias in ASD (24). Using ingroup and outgroup conditions with ecologic scenarios, the authors showed that intergroup bias was relatively attenuated in children with ASD compared to those with attention deficit hyperactivity disorder, learning disabilities, and intellectual disability. However, that study focused on pediatric population with developmental disorders and did not include individuals with typical development (TD). In addition, the sample size of participants with ASD was small (*n* = 9). Hence, intergroup bias that can be compared between individuals with ASD, especially adults, and their neurotypical counterparts remains unexplored.

This study investigated intergroup bias in adults with ASD and TD to fill this gap. We applied a third-party punishment paradigm with manipulation of group membership (ingroup/outgroup) in this endeavor, based on the prior investigations of intergroup bias on healthy subjects (2, 25, 26). In our experiment, participants observed a dictator game in which proposers could decide how to distribute the money, while receivers could only accept unconditionally (see Methods section for details). Participants were asked to play a role of the third party, and to further express their intention (or lack thereof) to punish the proposers by evaluating the reasonableness of the offers.

Previous studies have shown that empathy, which has been repeatedly reported to be altered in ASD (27, 28), plays a key role in generating intergroup bias (29, 30). In addition, recent research revealed that individuals with ASD showed atypical generosity because of their lower flexibility when switching decision rules and/or distinguishing between self's and other' perspectives (31). Thus, we hypothesized that participants with ASD would have less intergroup bias than those with TD. Particularly, we predicted that the punishment level in ASD would be less influenced by the affiliations of the proposer and recipient groups.

## Methods

### Participants

A total of 24 adults with ASD and 24 TD adults were enrolled in this study. We enrolled only male participants because of potential gender differences in the intergroup bias in punishing behavior (3, 32, 33). All participants were Japanese. Participants with ASD were recruited from a database of volunteers who were clinically diagnosed with ASD in the outpatient unit of the Showa University Karasuyama Hospital. The diagnostic procedure to identify individuals with ASD was the same as in our previous studies (34–36). Please see Supplementary Materials for details regarding participants.

Based on the previous studies on decision-making (37–39), we checked the participants' numeracy skills and understanding of numbers using a numeracy test. One participant with ASD was excluded from the analysis, because his score on the numeracy test was low than the overall average (>3 SD below the mean), suggesting that he did not have the basic numeracy skills necessary to understand the task. Thus, data obtained from 23 participants with ASD and 24 TD participants were analyzed (age: 20–45 years). There were no significant differences between the groups in age, current smoking status, and estimated full-scale intelligence quotient (IQ) levels [smoking status is reportedly associated with various types of decision-making (40)]. In total, 11 participants with ASD were administered the following psychotropic drugs: anxiolytics (*n* = 3), antidepressants (*n* = 5), antipsychotics (*n* = 2), antiepileptics (*n* = 1), sleep-inducing drugs (*n* = 5), and other psychotropic drugs (*n* = 3). All participants completed the Japanese version of the Autism Spectrum Quotient (AQ) test that includes items covering both social and non-social aspects of behavior and cognition (41, 42). The AQ was scored using the collapsed scoring system (41, 42). Higher scores indicated higher autistic traits.

This study was approved by the Committee on Medical Ethics of Kyoto University and the institutional review board of Showa University Karasuyama Hospital as well as conducted following The Code of Ethics of the World Medical Association. After a complete description of the study, written informed consent was obtained from all participants.

### Third-party punishment task

We modified a third-party punishment of the dictator game paradigm (43, 44).

One of the unique characteristics of human society is that people adhere to social norms and encourage altruistic behaviors (2, 21, 43). A key element of enforcing many social norms, such as food-sharing norms in hunter-gatherer societies (21, 45), is that people punish norm violators not only for direct transgressions against the punisher himself (i.e., second-party punishment) but also for norm violations against others (i.e., third-party punishment) (2, 21, 43). Third-party punishment is considered one of the decisive aspects of social norms in human society because third parties would punish norm violators altruistically, even though norm transgressions do not directly affect them (25, 43, 44). Crucially for this study, third-party punishment has been strongly shaped by intergroup bias (2, 21, 25, 26).

To investigate the third-party punishment, Fehr et al. (43) introduced the third-party punishment paradigm of the dictator game, in which proposers could decide how to distribute the money while receivers could only accept unconditionally (see Supplementary Materials for details regarding dictator game). To date, the paradigm has been used globally, irrespective of culture, and shown as a powerful tool for studying the mechanisms and individual difference factors of third-party punishment (4, 44). Furthermore, by modifying this well-established paradigm, previous studies have successfully estimated the intergroup bias objectively and quantitatively (26, 46). Thus, in this study, we used the third-party punishment paradigm of the dictator game to analyze intergroup bias.

Before starting the experiment, based on our previous study (38), the participants were interviewed about their social identities, including hometown (47, 48), sports team loyalty (1, 49), and political views (50, 51), the powerful sources of intergroup bias. As a cover story, the participants were instructed that they would be divided into groups based on interviews. Then, they were also instructed to grab the opportunity, as the third party, to punish anonymous Japanese players (their group members or other group members) in other rooms online. In reality, these players were not real people, and participants played against a computer programmed in advance.

**Figure 1:**
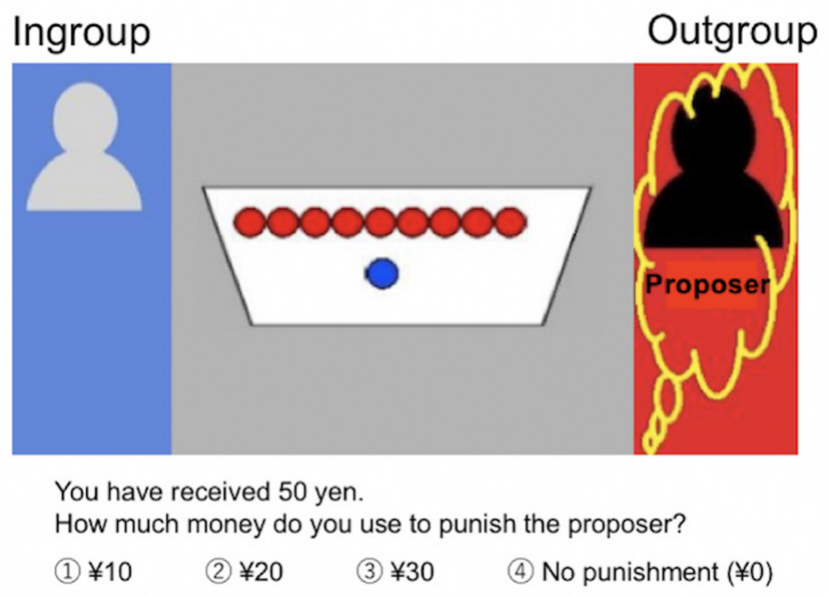
Third-party punishment task. Participants observed a dictator game in which proposers (yellow squiggles, red background) made offers to recipients (blue background). The proposer divided ¥100 between himself and the recipient who had to accept the proposal. The proposers and recipients were ingroup (gray) or outgroup (black) members. In this Figure, the amounts allocated to the proposer (outgroup) were ¥90 and ¥10 were allocated to the recipient (ingroup). At the beginning of each trial, participants received an endowment of ¥50 and were instructed to choose an amount [between ¥0 and ¥30 (in increments of ¥10)] to punish the proposers. Assigning ¥10 costs the participant ¥10 and the sanctioned proposer ¥30.

Participants observed a dictator game, in which proposers made offers to recipients. They were informed that the proposer divided ¥100 (about 1$) between himself and the recipient who had to accept the proposal. Based on previous studies on behavioral economics (52, 53), we established the amount of the initial allocation for the proposer at ¥100. The amounts of money allocated to the proposer and the recipient are indicated in Figure 1. Participants, the third party, evaluated the reasonableness of the offers and expressed their intention (or lack thereof) to punish the proposers. At the beginning of each trial, participants received an endowment of ¥50 and were instructed to choose an amount (between ¥0 and ¥30 [in increments of ¥10]) to punish the proposers. Assigning ¥10 costs the participant ¥10 and the sanctioned proposer ¥30. Based on previous studies (16, 53, 54), participants were told that endowments not used for punishment were exchanged for real money and paid to them (at the end of the experiment, we debriefed them about the purpose of the experiment and paid the maximum predefined participation fee).

To measure the participants' intergroup bias, based on the previous studies (2, 25), third parties (participants) were confronted with different combinations of proposer's and recipient's group affiliations: (i) the proposer was an ingroup member, whereas the recipient was an outgroup member (IN/OUT condition) or (ii) the proposer was an outgroup member, whereas the recipient was an ingroup member (OUT/IN condition). Comparing participants' punishment decisions between these two group situations reveals their intergroup bias (2, 25). To avoid habituation and increase the ecological validity, we also included the condition where both the proposer and recipient were ingroup members (IN/IN condition), which was not used for the main analysis.

Each condition consisted of 10 trials (the amounts of money allocated to the recipient were ¥50, ¥40, ¥30, ¥20, or ¥10, and each appeared twice). The presentation orders of conditions and allocated amounts to the recipient were randomized among participants. Then participants were instructed that there were no repeated interactions in the paradigm and all interactions were conducted in complete anonymity to control for reputation effects (2, 25, 53).

Participants practiced on a shorter version of the current task at least once and were corrected or instructed if any misunderstanding about how to play the task. The experiment was performed using E-Prime software (Psychology Software Tools, Inc., Pittsburgh, PA, USA).

### Statistical analyses

First, for the mean of the punishment amounts, a mixed analysis of variance (ANOVA) was used to examine group effects (TD vs. ASD), condition effects (IN/OUT vs. OUT/IN), and interaction of these factors.

Next, based on previous studies (2, 25), an intergroup bias score was calculated by subtracting mean punishment amounts in the IN/OUT condition from those in the OUT/IN condition. This score was used as an indicator of the strength of each participant's intergroup bias. High values on this intergroup bias score indicate that the participants' judge strongly differed for treating ingroup and outgroup members. Low scores indicate that the participant treated both ingroup and outgroup members equally. Thus, a higher intergroup bias score indicates a stronger intergroup bias.

We performed correlation analyses between the intergroup bias score and severity of clinical symptoms evaluated using the AQ (total and subscale scores) among the participants with ASD.

Statistical analyses were performed using SPSS 24 (IBM, Armonk, NY, USA). Results were considered statistically significant at *p* < 0.05 (two-tailed).

## Results

Demographic and clinical data are shown in Table 1. No significant differences were observed between the groups with regard to age, current smoking status, and estimated full-scale IQ levels.

**Table 1:** Demographic and clinical characteristics of participants.

	**TD group**	**ASD group**	**Statistics**
	**(*n* = 24)**	**(*n* = 23)**	***p***
Age (years, mean ± S.D.)	26.0 ± 6.9	29.0 ± 4.5	0.09^a^
Current smoker/non-smoker	3/21	3/20	0.96^b^
Estimated full-scale IQ (mean ± S.D.)	105.0 ± 9.7	107.0 ± 12.3	0.53^a^
AQ total (mean ± S.D.)	15.7 ± 6.7	34.1 ± 5.7	<0.01^a^
Social skill (mean ± S.D.)	2.3 ± 2.3	7.3 ± 2.6	<0.01^a^
Attention switching (mean ± S.D.)	3.5 ± 1.5	8.2 ± 1.4	<0.01^a^
Attention to detail (mean ± S.D.)	5.0 ± 2.2	5.1 ± 2.3	0.84^a^
Communication (mean ± S.D.)	2.0 ± 2.2	7.2 ± 1.8	<0.01^a^
Imagination (mean ± S.D.)	3.0 ± 1.6	6.3 ± 1.8	<0.01^a^

a Two-sampled t-test.

b Two-tailed chi-squared test.

ASD, autism spectrum disorder; AQ, Autism Spectrum Quotient; IQ, Intelligence Quotient; TD, typical development.

**Figure 2:**
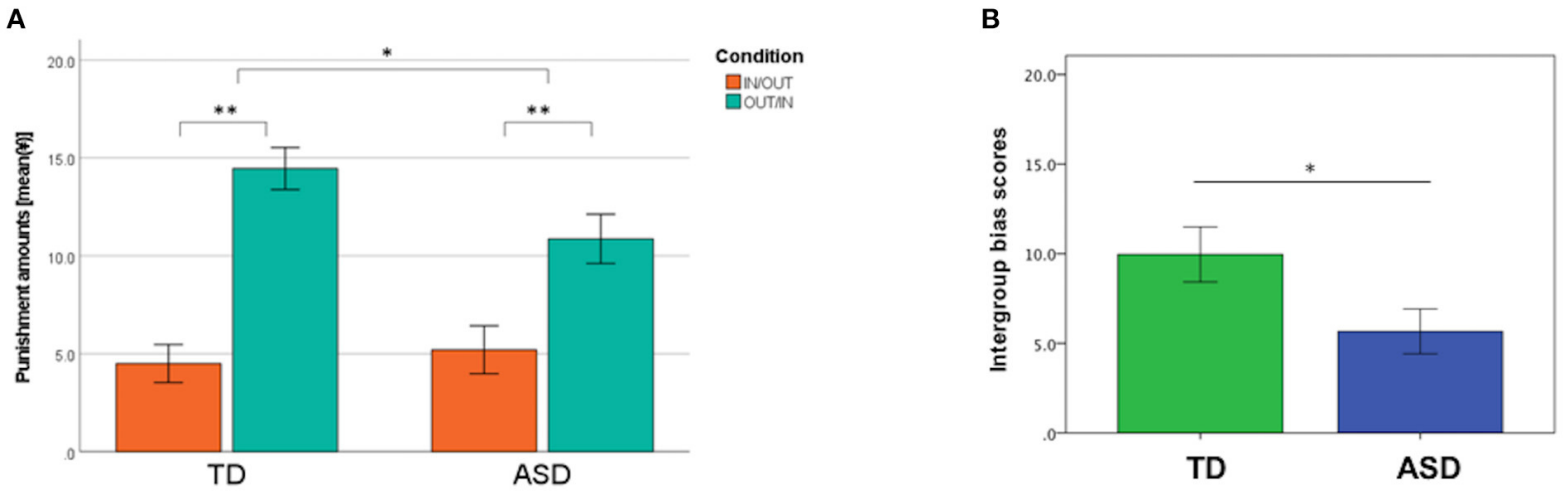
Behavioral data in the third-party punishment task. **(A)** The mean amounts of punishment in the IN/OUT (the proposer was an ingroup member, whereas the recipient was an outgroup member) and OUT/IN (the proposer was an outgroup member, whereas the recipient was an ingroup member) conditions in the typical development (TD) and autism spectrum disorder (ASD) groups. The 2 × 2 mixed analysis of variance revealed the presence of a group × condition interaction effect (*F* = 4.65, *p* = 0.036). **(B)** Intergroup bias scores in the TD and ASD groups. *p < 0.05, **p < 0.01. These *p*-values were not corrected for multiple testing. Error bars indicate ± standard errors.

Figure 2 shows the mean amounts of punishment in the IN/OUT and OUT/IN conditions in TD and ASD groups, respectively. A 2 × 2 mixed ANOVA showed that the main effect of the group was not significant (*F*_1, 45_ = 1.32, *p* = 0.26, $\eta_{p}^{2}$ = 0.028). However, we found a significant main effect of the condition (*F*_1, 45_ = 61.64, *p* < 0.01, $\eta_{p}^{2}$ = 0.58). Furthermore, a significant group × condition interaction (*F*_1, 45_ = 4.65, *p* = 0.036, $\eta_{p}^{2}$ = 0.094) was observed. The mean amounts of punishment in the OUT/IN condition were significantly higher than those in the IN/OUT condition in both groups (TD, *p* < 0.01, ASD, *p* < 0.01). The mean amounts of punishment in the OUT/IN condition in the TD group were higher than those in the ASD group (*p* = 0.035). However, this difference was not significant after the Bonferroni correction for multiple testing [*p* corrected = 0.0125 (0.05/4)]. No significant differences were observed in the amounts of punishment in the IN/OUT condition between groups (*p* = 0.65).

Next, an intergroup bias score was estimated for each participant based on their chosen behavior. The intergroup bias score was significantly lower in the ASD group than that in the TD group (TD 9.96 ± 7.52, ASD 5.67 ± 6.00, *p* = 0.036). Considering that 11 participants with ASD took psychotropic drugs, the intergroup bias score of participants with ASD who were not taking psychotropic drugs (*n* = 12) was compared with that of TD participants. The analysis did not materially change the result; the intergroup bias score among participants with ASD who were not taking psychotropic drugs was found to be significantly lower than that of the TD group (TD 9.96 ± 7.52, ASD [without psychotropic drugs] 5.03 ± 3.61, *p* = 0.012). No significant difference was found in the intergroup scores between the ASD participants with and without psychotropic drugs (ASD [with psychotropic drugs] 6.36 ± 7.99, ASD [without psychotropic drugs] 5.03 ± 3.61, *p* = 0.62).

Thereafter, correlation analyses were performed between the intergroup bias score and clinical symptom severity among participants with ASD. We found no significant relationship between the intergroup score and clinical symptom severity (all, *p* > 0.20). As ASD characteristics form a continuum that extends to the characteristics of the typical population (34, 41), correlation analyses between the intergroup bias score and AQ (total and subscale scores) were also performed among the participants (both the ASD and TD groups) and no significant correlations were observed (all, *p* > 0.18).

### Additional analyses

To explore the data in more detail, we performed the following analyses.

### Effects of trials on intergroup bias

**Figure 3:**
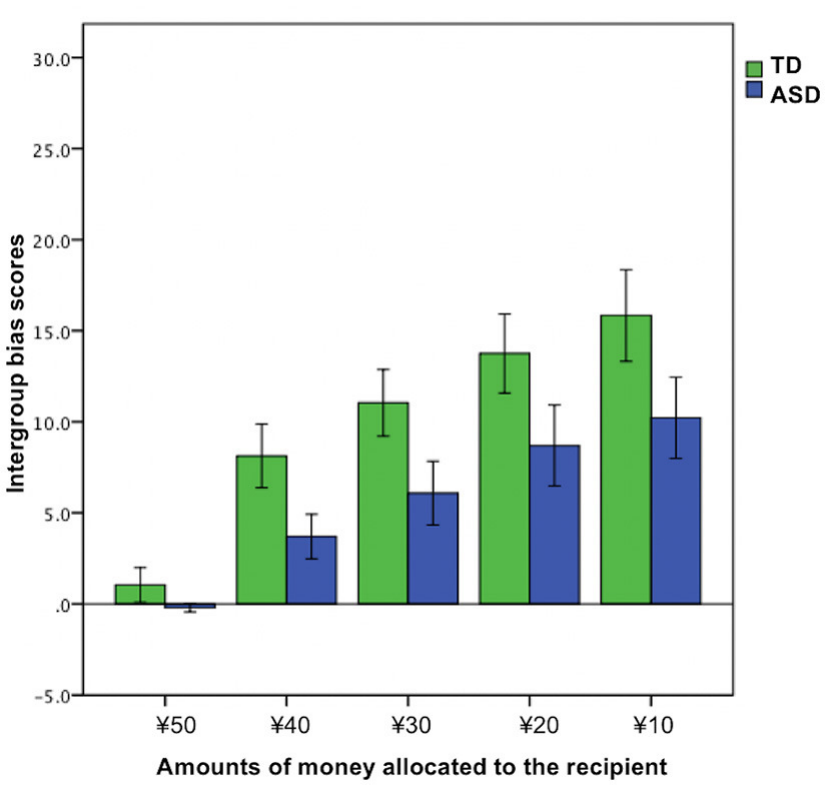
Trial effects on intergroup bias. Error bars indicate ± standard errors.

To explore the effects of trials (the amounts of money allocated to the recipient were ¥50, ¥40, ¥30, ¥20, or ¥10) on intergroup bias, we ran a 2 × 5 mixed ANOVA for the intergroup bias score based on group (TD vs. ASD) × trial [¥50 vs. ¥40 vs. ¥30 vs. ¥20 vs. ¥10 (the amounts of money allocated to the recipient)]. Significant main effects were observed in the group (*F*_1, 45_ = 4.59, *p* = 0.038, $\eta_{p}^{2}$ = 0.093) and trial (*F*_2.53, 113.84_ = 29.34, *p* < 0.01, $\eta_{p}^{2}$ = 0.39). However, we did not find a significant group × trial interaction (*F*_2.53, 113.84_ = 0.90, *p* = 0.43, $\eta_{p}^{2}$ = 0.02) (Figure 3).

### Punishing behavior in IN/IN condition

**Figure 4:**
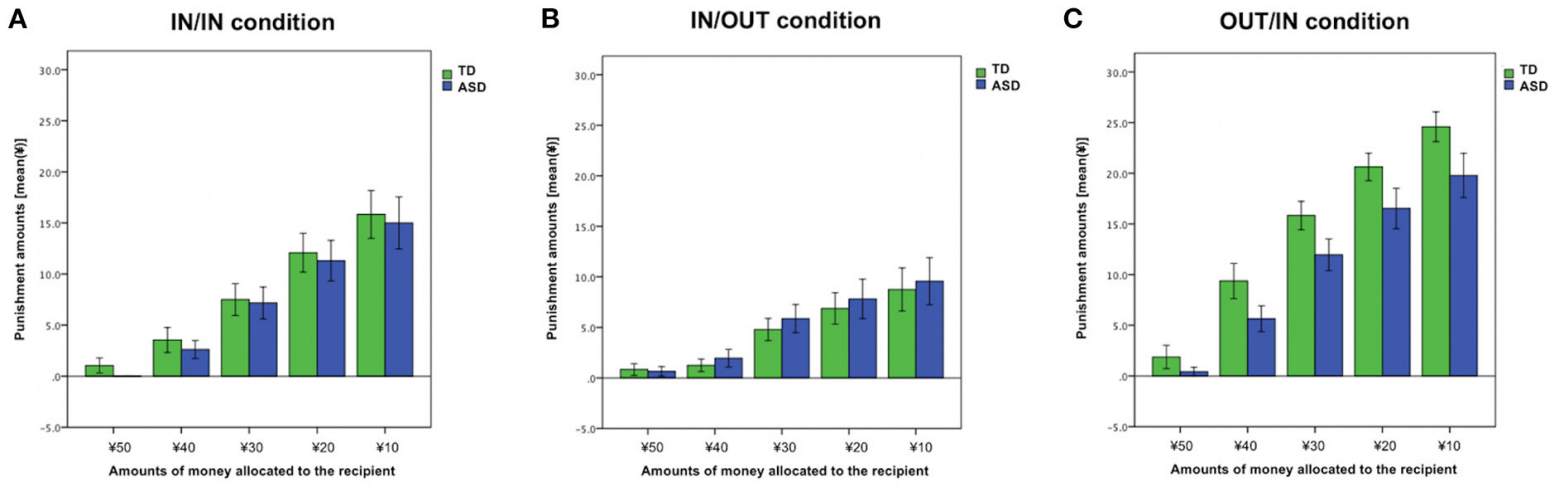
Punishment amount across each trial. **(A)** IN/IN (both the proposer and recipient were ingroup members) condition. **(B)** IN/OUT (the proposer was an ingroup member, whereas the recipient was an outgroup member) condition. **(C)** OUT/IN (the proposer was an outgroup member, whereas the recipient was an ingroup member) condition. Error bars indicate ± standard errors.

For the punishment amounts in IN/IN condition, a 2 × 5 mixed ANOVA was performed to examine group effects (TD vs. ASD), trial effects [¥50 vs. ¥40 vs. ¥30 vs. ¥20 vs. ¥10 (the amounts of money allocated to the recipient)], and interaction of these factors. The main effect of the trial was observed (*F*_1.72, 77.39_ = 54.34, *p* < 0.01, $\eta_{p}^{2}$ = 0.55). However, we did not find a group effect nor group × trial interaction (both, *p* > 0.66) (Figure 4A). Punishment amounts in IN/OUT and OUT/IN conditions are also shown in Figures 4B,C.

## Discussion

To the best of our knowledge, this is the first study to investigate an intergroup bias among the adults with ASD using a third-party punishment of the dictator game paradigm.

Indeed, our TD group showed a clear intergroup bias in the current task. The mean amounts of punishment in the OUT/IN condition were significantly higher than those in the IN/OUT condition. These results are in accordance with the previous studies (2, 25), and support the notion that intergroup bias is inevitable during social communication; and that such in/out group-oriented behaviors are essential in human society (1).

As predicted, the intergroup bias was attenuated in the ASD group compared with the TD group. Recently, Vaucheret Paz et al. (24) investigated this tendency in children with developmental disorders using two types of videos showing football games. In one video (video-1), a football player from the participant's country scored a goal with his hand. In another video (video-2), a player from another country did the same against the participant's country. The ASD group showed negative feelings in both videos; however, the attention deficit hyperactivity disorder, learning disabilities, intellectual disability groups showed positive opinions in video-1 and negative in video-2. These results suggest that the intergroup bias is less in children with ASD. Our results are consistent with these previous experimental findings and demonstrate that the attenuated intergroup bias can be also observed in adults with ASD in addition to children with ASD.

The findings of the current study raise a question: why do individuals with ASD show a lesser intergroup bias? This can be explained due to altered empathy in individuals with ASD. Empathy is a psychological capacity that facilitates people to understand and respond to the emotional experiences of others (55, 56). It can play an important role in interpersonal communication and refined social functioning (55, 56). People have been reported to show more automatic empathy toward ingroup members as compared to outgroup members (29, 30). Furthermore, previous studies showed that third-party punishment was modulated by individual differences in trait empathy among healthy participants (26). It is widely reported that individuals with ASD show atypical behaviors when there is a need to empathize or take perspective from other people (27, 28). Thus, individuals with ASD may choose to follow rules rather than favoring ingroup members or hating outgroup members. As an alternative interpretation, a lower intergroup bias in individuals with ASD may be explained by their impaired cognitive flexibility defined as an ability to switch between (or think about) different/multiple concepts and choices simultaneously (34, 52). Previous research has shown that atypical generosity in ASD may partly stem from lower flexibility when switching decision rules and/or distinguishing self/other perspectives (31). These experimental results lead to the idea for the current study that individuals with ASD may have difficulties in flexibly changing their decision based on group membership. Previous functional magnetic resonance imaging studies on healthy participants have revealed that several brain regions, including the dorsomedial prefrontal cortex, lateral prefrontal cortex, anterior cingulate cortex, and temporoparietal junction, are involved in decision-making under situations involving group membership (21, 51, 57). Notably, these brain areas are often altered in individuals with ASD (58–62). Thus, future neuroimaging studies including the group membership condition should provide useful information for mechanisms of altered decision-making among individuals with ASD when considering group membership. Furthermore, as ASD characteristics lie on a continuum that extends into the typical population, these findings will offer significant insights into generating intergroup bias in human society.

Additional analyses of the trial effects on intergroup bias suggested two pieces of useful information for the better understanding of decision-making under situations involving a group membership in ASD. First, the ASD group did not show intergroup bias when the allocation was fair (the amounts of money allocated to the recipient was ¥50); that is, participants with ASD were completely unbiased when the outgroup behaved fairly unlike TDs, although both the TD and ASD groups tended to engage in more intergroup bias as the allocation of the proposer became increasingly unfair. Second, as shown in Figure 3, the punishing behaviors were numerically less biased in the ASD group than the TD group almost equally across all the levels of fairness. This observation deepens our understanding of the specific altered behavioral patterns observed in ASD, and hence, should be investigated further in future research using various scenarios and a wide range of trials.

Our findings have implications on the practical, social, and economic functioning of individuals with ASD because the intergroup bias is highly prevalent in real life (1–4). Intergroup bias improves group functioning and allows an individual to fit into a group (1, 38, 49). For example, it can provide internal safety and security against outside threats, and this bias can further prompt beneficial exchanges with ingroup members as well as mutual/social supports (3, 38). Such intergroup bias develops an ability to distinguish between the behaviors of the ingroup and outgroup members. Conversely, intergroup bias prompts numerous negative human deeds, such as excessive competition, discrimination, and violent protest (1, 63, 64). Thus, the attenuated intergroup bias in ASD might be useful in avoiding intergroup conflict in our social lives. Furthermore, this tendency in individuals with ASD would be helpful in fostering diversity, equity, inclusion, and belonging in many situations, including education, employment, and exercise of public functions. Similar to previous studies (13–16), our findings contribute to a better understanding of ASD by showing a valuable strength as well as difficulty in decision-making of individuals with ASD.

Understanding the heterogeneity of symptom expression in ASD is key for better comprehending its underlying neurobiological mechanisms and establishing precise treatment strategies (14–16). Behavioral economic tools can help elucidate the existing symptomatology of ASD or inform the development of new mediating markers and personalization of treatment (15–18). No significant correlations were found between the levels of intergroup bias and AQ scores in the present study. This might be due to the sample size for detecting the possible correlations. This is one of the major limitations of the current study and should be overcome in future studies by including a larger number of participants.

The laboratory task in the current study has the advantage of creating a simple hypothesized intergroup context. It can minimize the effects of participants' various interpretations of the experimental setup on their decisions (which are oftentimes difficult to control without hypothesized conditions) (65, 66). Such context-controlled decision situations have been reported to mitigate the impact of the internal stimulus and thus curtail distortion of experimental results (65, 66). However, decision situations presented in our laboratory experiments are abstract and somewhat unparalleled in real-life situations, and thus, generalizing these data should cautiously made. Furthermore, to some extent, due to such a laboratory hypothesized setting, participants may become more generous because of “windfall gains,” whereas in the real-life situation (outside the lab), participants would become less generous, because they would focus more on obtaining their monetary gain (rather than investigating hypothesized money to other people) (65, 67). Thus, our findings may also warrant caution in this context, although previous studies have demonstrated that the results of economic game tasks in the laboratory fairly were associated with real-world behaviors (65). In effect, more refined, ecological valid studies are required to further explore human decision-making that can offer important complementary insights.

In addition to the above-mentioned issues, this study has several limitations. First, we did not perform the experiment using a real social group; rather, we created a situational setting for participants in which they were facing other players from the ingroup/outgroup using a cover story. Nevertheless, the post-task interview confirmed that participants were led to believe that they were playing with real people and that their decisions had real consequences. Consistent with the previous studies (43), the punishment amounts of the participants further increased the distribution norms were violated (the lesser the amounts of money were allocated to the recipient). Furthermore, none of the participants showed >2 SD below the mean regarding reaction time (an extremely fast reaction time implies poor decision quality [e.g., (53, 68)]), which supports our contention that all the participants made substantial efforts to tackle our task. Thus, we believe that our findings are useful for understanding decision-making under situations involving a group membership in ASD. Second, the current task did not include conditions where both the proposer and recipient were outgroup members (OUT/OUT condition) and options other than punishing the proposers (e.g., compensating the recipients). Therefore, we cannot differentiate whether participants preferred punishing outgroup proposers or protecting ingroup recipients to ameliorate unfair distributions. Third, based on previous studies (53, 69, 70), we carefully selected the color used in our task. However, the chosen color might affect the performance of participants. Fourth, the participants' asset size and financial sense can influence their choice behavior (65). These issues should be overcome in future studies using a real social group and tasks including more conditions by controlling potential confounding factors.

Fifth, this study has a small sample size. No significant differences have been found after Bonferroni correction in the mean amount of the punishment in the OUT/IN condition (as well as IN/OUT condition) between the TD and ASD groups. In line with previous studies (4), the results suggest that the interaction effect (difference of the intergroup bias) is driven by both OUT/IN and IN/OUT conditions. However, such null findings should be considered in the context of a lower power for detecting significant differences. Sixth, nearly half of participants with ASD were administered psychotropic medication, indicating that the possibility of a medication effect cannot be excluded. Unfortunately, medicated participants with ASD in our study were administered different types of psychotropic drugs. This has prevented any further analysis of medication effects on behavioral results. However, the intergroup bias score of participants with ASD who were not taking psychotropic drugs was also significantly attenuated compared with that of the TD participants. Seventh, our sample comprised male participants only. Previous studies have shown that women identify with their ingroup more strongly than men (33) and women show ingroup favoritism regardless of dependence on the ingroup, whereas men show this tendency when they depend on ingroup members for outcomes (32). Thus, it is crucial to include female participants for generalizing our current findings that should be pursuit in the future. Finally, our ASD sample consisted of high functioning individuals with ASD only. To replicate and strengthen our findings, further studies are required to include more ASD individuals with female sex, diverse IQ levels, and individuals who are not under medication.

Despite these limitations, the current results suggest that intergroup bias is attenuated in individuals with ASD. Our findings address the practical implications of socioeconomic behaviors of individuals with ASD and contribute to a better understanding of altered decision-making in them.

## Data availability statement

The raw data supporting the conclusions of this article will be made available by the authors, without undue reservation.

## Ethics statement

The studies involving human participants were reviewed and approved by the Committee on Medical Ethics of Kyoto University and the institutional review board of Showa University Karasuyama Hospital. The patients/participants provided their written informed consent to participate in this study.

## Author contributions

ST, TI, R-iH, MN, HT, NK, and JF designed research. ST, TI, and JF participated in the data acquisition. YA, HO, MN, NK, and JF were in charge of the clinical assessment. CQ, ST, and JF analyzed data and managed the literature searches. TI, YA, HO, R-iH, MN, HT, and NK helped with interpretation of data. CQ, ST, TI, YA, HO, R-iH, MN, HT, NK, and JF wrote the paper. All authors have made intellectual contribution to the work and approved the final version of the manuscript for submission.

## Funding

This work was supported by grants-in-aid for Young Scientists (17K16398 and 20K16654) and Scientific Research C (17K10326 and 21K07544) from the Ministry of Education, Culture, Sports, Science and Technology of Japan (MEXT); a grant from SENSHIN Medical Research Foundation; and Intramural Research Grant (2-7) for Neurological and Psychiatric Disorders of NCNP. A part of this study is the result of the Joint Usage/Research Program of Medical Institute of Developmental Disabilities Research, Showa University. These agencies had no further role in the study design, collection, analysis, and interpretation of data; the writing of the report; or the decision to submit the paper for publication.

## Conflict of interest

The authors declare that the research was conducted in the absence of any commercial or financial relationships that could be construed as a potential conflict of interest.

## Publisher's note

All claims expressed in this article are solely those of the authors and do not necessarily represent those of their affiliated organizations, or those of the publisher, the editors and the reviewers. Any product that may be evaluated in this article, or claim that may be made by its manufacturer, is not guaranteed or endorsed by the publisher.
