# Supplementary material for: Intergroup bias in punishing behaviors of adults with autism spectrum disorder
Source: Front Psychiatry. 2022 Aug 19;13:884529. doi: 10.3389/fpsyt.2022.884529 (PMC9437315; doi:10.3389/fpsyt.2022.884529)
Supplement: Supplementary file 1 [file Table_1.DOCX]

Supplementary Material

**Supplementary Methods**

***Participants***

Twenty-four adults with ASD and 24 typical development (TD) adults were enrolled in this study. Participants with ASD were recruited from a database of volunteers clinically diagnosed with ASD in the outpatient unit of the Showa University Karasuyama Hospital. The diagnostic procedure to identify individuals with ASD was the same as in our previous studies (1-3). At least three experienced psychiatrists (Y.Y.A., H.O., M.N., N.K., and J.F.) and a clinical psychologist assessed all patients using the criteria from the Diagnostic and Statistical Manual of Mental Disorders, fourth edition text revision (DSM-IV-TR). The assessment comprised of participant interviews about developmental history, present illness, life history, and family history. Patients were also instructed to bring suitable informants who had known them during early childhood. The whole process required approximately 3 h. A diagnosis of ASD was made only when a consensus was achieved between the psychiatrists and clinical psychologist. At the time of testing, an experienced psychiatrist (J.F.) evaluated psychiatric comorbidity using the Structured Clinical Interview for DSM-IV Axis I Disorders (SCID). No participants with ASD met the diagnostic criteria for substance use disorder, bipolar disorder, or schizophrenia. Individuals with TD were recruited through advertisements and acquaintances and did not meet the criteria for any psychiatric disorders based on the evaluation by an experienced psychiatrist using SCID. No participants (TD or ASD) had any history of head trauma, serious medical or surgical illness, or substance abuse.

Based on the previous studies on decision-making (4-6), we checked the participants’ numeracy skills and understanding of numbers using a numeracy test. One participant with ASD was excluded from the analysis, because his score on the numeracy test was low than the overall average (> 3 SD below the mean), suggesting that he did not have the basic numeracy skills necessary to understand the task. Thus, data obtained from 23 participants with ASD and 24 TD participants were analyzed (age: 20–45 years). The ASD diagnosis of 19 participants was further supported by the Autism Diagnostic Observation Schedule (ADOS)-1 or -2 (7, 8). Three participants did not satisfy the diagnostic criteria of ASD per the ADOS, and ADOS could not be performed for one participant due to the participant’s schedule. However, the ADOS classification is not a clinical diagnosis (i.e., based on standardized DSM/ICD) and, although it may contribute to diagnosis, it cannot replace the full systematic assessment of ASD by specialized clinicians. Thus, based on previous studies (e.g., 9, 10), we included the four participants who were clinically diagnosed with ASD by experienced psychiatrists (Y.Y.A., H.O., M.N., N.K., and J.F.) who have been practicing for more than 10 years.

The intelligence quotient (IQ) scores of all participants with ASD had been evaluated before the study using either the Wechsler Adult Intelligence Scale-Third Edition or the WAIS-Revised. All participants with ASD had a full-scale IQ score of >80 and were therefore considered high functioning. The IQ scores of participants with TD were estimated using the Japanese version of the National Adult Reading Test based on previous studies (11, 12).

***Dictator Game***

Prosociality can often be costly, and the perceived cost is a key helping determinant (13). Examples of real-world costly prosocial acts include charitable giving (which involves sacrificing money) and volunteering (which involves sacrificing time) for the benefit of others (13, 14). In laboratory examinations, costly prosocial preferences (unselfish behavior) can be assessed using the dictator game, where one player (the dictator) decides how to split a fixed amount of money with an anonymous recipient who must unconditionally accept this (15, 16). Previous behavioral economics studies consistently demonstrate that individuals share substantial amounts of their endowments with anonymous recipients in the dictator game (16, 17).

**Supplementary References**

1. Fujino J, Tei S, Itahashi T, Aoki Y, Ohta H, Kubota M. et al. Need for closure and cognitive flexibility in individuals with autism spectrum disorder: A preliminary study. *Psychiatry Res.* (2019) 271:247-52.

2. Fujino J, Tei S, Itahashi T, Aoki YY, Ohta H, Izuno T et al. A single session of navigation-guided repetitive transcranial magnetic stimulation over the right anterior temporoparietal junction in autism spectrum disorder. *Brain Stimul.* (2021) 14:682-4.

3. Kubota M, Fujino J, Tei S, Takahata K, Matsuoka K, Tagai K et al. Binding of dopamine D1 receptor and noradrenaline transporter in individuals with autism spectrum disorder: A PET Study. *Cereb Cortex.* (2020) 30:6458-68.

4. Fujino J, Tei S, Hashimoto RI, Itahashi T, Ohta H, Kanai C et al. Attitudes toward risk and ambiguity in patients with autism spectrum disorder. *Mol Autism.* (2017) 8:45.

5. Fujino J, Tei S, Itahashi T, Aoki YY, Ohta H, Kubota M et al. Role of the right temporoparietal junction in intergroup bias in trust decisions. *Hum Brain Mapp.* (2020) 41:1677-88.

6. Pushkarskaya H, Tolin D, Ruderman L, Kirshenbaum A, Kelly JM, Pittenger C et al. Decision-making under uncertainty in obsessive–compulsive disorder. *J Psychiatr Res.* (2015) 69:166-73.

7. Gotham, K., Risi, S., Pickles, A., Lord, C. The Autism Diagnostic Observation Schedule: revised algorithms for improved diagnostic validity. *J Autism Dev Disord.* (2007) 37:613–27.

8. Gotham, K., Pickles, A., Lord, C. Standardizing ADOS scores for a measure of se- verity in autism spectrum disorders. *J Autism Dev Disord.* (2009) 39:693–705.

9. De Martino B, Harrison NA, Knafo S, Bird G, Dolan RJ. Explaining enhanced logical consistency during decision making in autism. *J Neurosci.* (2008) 28:10746-50.

10. Itahashi T, Fujino J, Sato T, Ohta H, Nakamura M, Kato N et al. Neural correlates of shared sensory symptoms in autism and attention-deficit/hyperactivity disorder. *Brain Commun.* (2020):fcaa186.

11. Matsuoka K, Uno M, Kasai K, Koyama K, Kim Y. Estimation of premorbid IQ in individuals with Alzheimer’s disease using Japanese ideographic script (Kanji) compound words: Japanese version of National Adult Reading Test. *Psychiatry Clin Neurosci.* (2006) 60:332-9.

12. Tei S, Fujino J, Itahashi T, Aoki Y, Ohta H, Kubota M et al. Egocentric biases and atypical generosity in autistic individuals. *Autism Res.* (2019) 12:1598-608.

13. Ferguson E, Zhao K, O’Carroll RE, Smillie LD. Costless and costly prosociality: Correspondence among personality traits, economic preferences, and real-world prosociality. *Soc Psychol Personal Sci.* (2019) 10:461-71.

14. Böckler A, Tusche A, Singer T. The structure of human prosociality: Differentiating altruistically motivated, norm motivated, strategically motivated, and self-reported prosocial behavior. *Soc Psychol Personal Sci.* (2016) 7:530-41.

15. Forsythe R, Horowitz JL, Savin NE, Sefton M. Fairness in simple bargaining experiments. *Games Econ Behav.* (1994) 6:347-69.

16. Franzen A, Pointner S. The external validity of giving in the dictator game. *Exp Econ.* (2013) 16:155-69.

17. Engel C. Dictator games: A meta study. *Exp Econ*. (2011) 14:583-610.
